# Association between red blood cell distribution width and all-cause mortality in unselected critically ill patients: Analysis of the MIMIC-III database

**DOI:** 10.3389/fmed.2023.1152058

**Published:** 2023-03-30

**Authors:** Shixuan Peng, Wenxuan Li, Weiqi Ke

**Affiliations:** ^1^Department of Oncology, Graduate Collaborative Training Base of The First People’s Hospital of Xiangtan City, Hengyang Medical School, University of South China, Hengyang, Hunan, China; ^2^Department of Anesthesiology, The First People’s Hospital of Yueyang, Yueyang, Hunan, China; ^3^Department of Anesthesiology, The First Affiliated Hospital of Shantou University Medical College, Shantou, Guangdong Province, China

**Keywords:** red blood cell distribution width, unselected adult patients, all-cause mortality, cox proportional hazards regression, K-M survival curves

## Abstract

**Background:**

Although red cell distribution width (RDW) is widely observed in clinical practice, only a few studies have looked at all-cause mortality in unselected critically ill patients, and there are even fewer studies on long-term mortality. The goal of our study was to explore the prediction and inference of mortality in unselected critically ill patients by assessing RDW levels.

**Methods:**

We obtained demographic information, laboratory results, prevalence data, and vital signs from the unselected critically ill patients using the publicly available MIMIC-III database. We then used this information to analyze the association between baseline RDW levels and unselected critically ill patients using Cox proportional risk analysis, smoothed curve fitting, subgroup analysis, and Kaplan–Meier survival curves for short, intermediate, and long-term all-cause mortality in unselected critically ill patients.

**Results:**

A total of 26,818 patients were included in our study for the final data analysis after screening in accordance with acceptable conditions. Our study investigated the relationship between RDW levels and all-cause mortality in a non-selected population by a smoothed curve fit plots and COX proportional risk regression models integrating cubic spline functions reported results about a non-linear relationship. In the fully adjusted model, we found that RDW values were positively associated with 30-day, 90-day, 365-day, and 4-year all-cause mortality in 26,818 non-selected adult patients with HRs of 1.10 95%CIs (1.08, 1.12); 1.11 95%CIs (1.10, 1.13); 1.13 95%CIs (1.12, 1.14); 1.13 95%CIs (1.12, 1.14).

**Conclusion:**

In unselected critically ill patients, RDW levels were positively associated with all-cause mortality, with elevated RDW levels increasing all-cause mortality.

## 1. Introduction

Several modern studies have shown that red blood cell distribution width (RDW) is a blood sampling assay used to determine the degree of variation in the volume of red blood cells in peripheral circulation. Red blood cell distribution width is a measure of the dispersion of red blood cell volume in terms of the width of the histogram distribution of red blood cells compared to their height and is included in blood cell analysis (1). Previously, RDW has been used for the diagnosis and differential diagnosis of different types of anemia (2). Red blood cell distribution width values are often seen in cases of nutrient deficiency, hemolysis, and anemia, and have been used to diagnose and classify anemia from various causes (3–5). An increase in RDW values is usually associated with an increase in the rate of erythrocyte proliferation. Because naive cells, such as reticulocytes, formed during the proliferation of red blood cells are larger than mature red blood cells, their presence leads to an increase in the width of the red blood cell distribution. However, it is important to note that an increase in the width of the erythrocyte distribution can also be caused by other factors such as inflammation, anemia and malnutrition (6).

Red cell distribution width has been studied in a variety of cohorts, but the majority of earlier research has focused on one or two specific blood biochemical indicators in a chosen population of patients with a single disease (e.g., renal disease, cardiovascular diseases, cerebral infarction, acute myeloid leukemia, Hodgkin Lymphoma.) or a particular risk group (e.g., emergency, intensive care unit, patients on specific medications) (7–11). Red cell distribution width, a clinical indication, has been proven to predict the prognosis of many diseases and has been researched about lengthened hospital stays, according to earlier research (12, 13). Although red cell distribution width is frequently observed in clinical practice, few studies have looked at short-, medium-, and long-term all-cause mortality in adult unselected critically ill patients. In addition, aside from studies of RDW and mortality in patients with specific diseases, the overall mortality of RDW and adult unselected critically ill patients is unknown, and studies of long-term mortality are even more scarce.

The goal of our study was to explore the association between red cell distribution width (RDW) levels and all-cause mortality in all unselected critically ill patients. To determine if RDW levels were independently associated with 30-day, 90-day, 365-day, and 4-year all-cause mortality among unselected critically ill patients, this study was designed.

## 2. Materials and method

### 2.1. Data source

The Critical Care III Version 1.4 (Mimic-IIIV.1.4) database was developed by Philips Healthcare, the Institutional Review Boards of Beth Israel Deaconess Medical Center (BIDMC, Boston, MA, United States) and the Massachusetts Institute of Technology (MIT, Cambridge, MA, United States). Included is information on more than 50,000 patients admitted to various ICUs (Intensive Care Units) at Boston from 2001 to 2012 (14). The database includes demographics, vital signs, patient comorbidities, biochemical indicators, laboratory tests, fluid balance, and vital status, with physiological data obtained from hourly tests by bedside monitors validated by ICU nurses and later assessed in writing by storage specialists during the appropriate time period. The use of the data in the database is provided by clinicians, data scientists, and IT staff. The use of the database is non-human subject experiments, does not require individual patient consent, and does not cause harm to the patient (14, 15). Users must pass the database test and be approved by the MIMIC-III database manager in order to be eligible to register and use the database. After passing the “Protecting Human Research Participants” training course on the website of the National Institutes of Health (NIH), author Weiqi Ke was approved to extract data from the database for this cohort study. Database for this cohort study (Record ID: 40171761).

### 2.2. Study design

Our retrospective cohort study involved 26,818 unselected critically ill patients and was carried out between 2001 and 2008. The clinical data from these patients was typical of regional critical care based on knowledge from multicenter clinical trials. With the intention of examining the relationship between RDW levels and short-, medium-, and long-term all-cause mortality in unselected critically ill patients, baseline RDW levels were employed as independent goal variables.

### 2.3. Study sample

Our study population was unselected critically ill patients. The criteria for inclusion were (1) patient’s age > 18 years; (2) patients who are listed in the accessible MIMIC-III database (more than 50,000 patients).

We excluded (1) patients under the age of 18; (2) patients with Dbsource = metavision; (3) patients with missing baseline RDW values at ICU admission.

**Figure 1:**
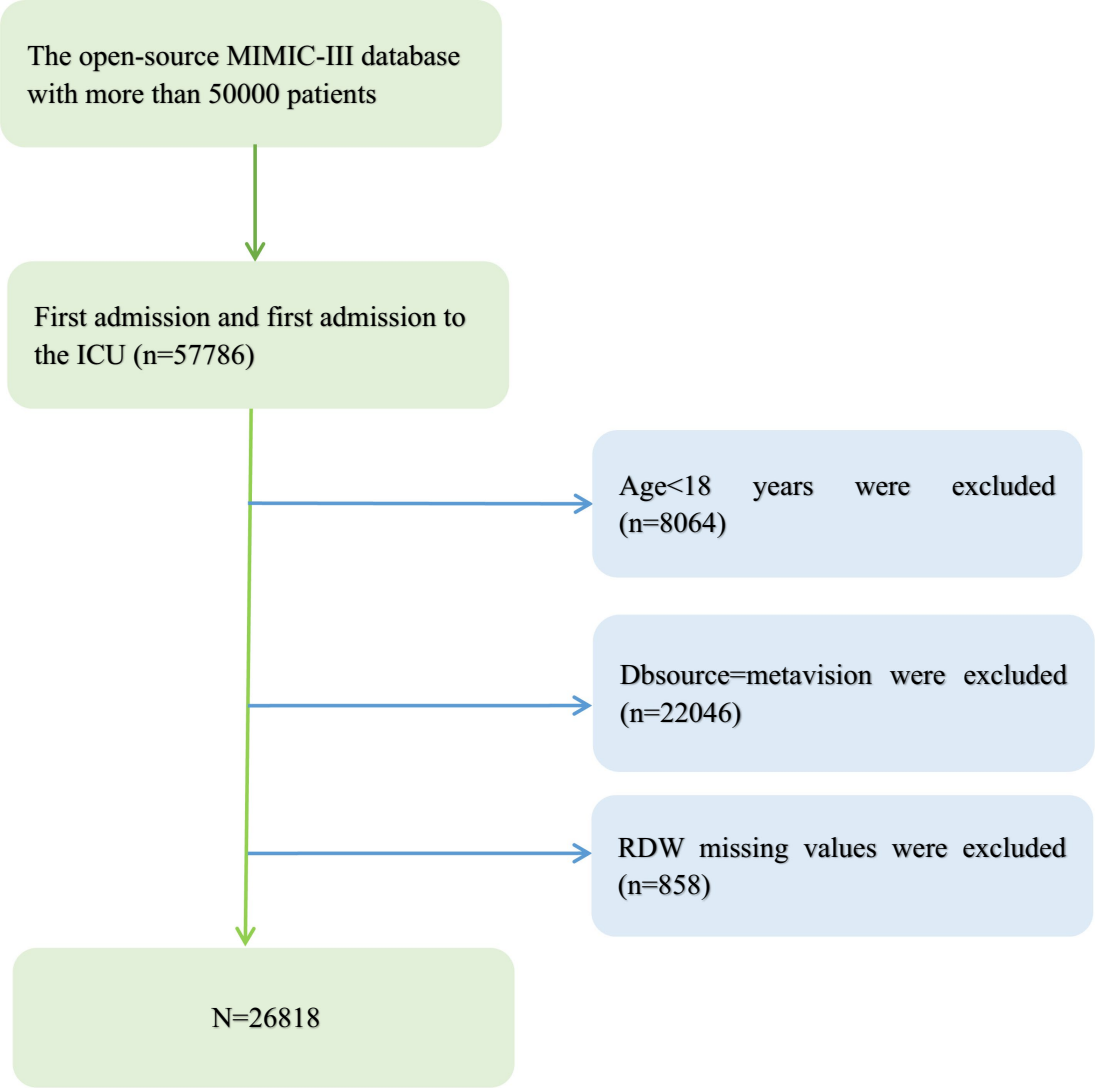
Flowchart of patient selection.

Since our RDW missing values are less than 3%, we do not need to perform multiple interpolation (see Figure 1).

### 2.4. Variables

We set the RDW value as a continuous variable for this study, with all-cause mortality recorded as a dichotomous variable and the following variables adjusted for in our study.

The following variables were used to construct the fully adjusted model: (1) Continuous variables (obtained at baseline): age; heart rate; systolic blood pressure (SBP); temperature; pulse oxygen saturation (SPO2); diastolic blood pressure (DBP); respiratory rate; anion gap; albumin level; blood urea nitrogen (Bun) level; Platelet level; sodium level; hemoglobin level; hematocrit level; glucose level; potassium level; creatinine level; bicarbonate level; Phosphate level, Magnesium level, Serum calcium level, the Simplified Acute Physiology Score II (SAPS II); the Sequential Organ Failure Assessment (SOFA) score; and the Elixhauser-van Walraven Comorbidity Index (EVCI); red blood cell (RBC) count; red blood cell distribution width (RDW) level; white blood cells (WBC) count. (2) Dichotomous variable: gender; insurance; admission type; cardiac arrhythmias; valvular disease; pulmonary circulation; congestive heart failure; peripheral vascular; hypertension; chronic pulmonary; diabetes uncomplicated; diabetes complicated; hypothyroidism; renal failure; liver disease; coagulopathy; blood loss anemia; deficiency anemias.

### 2.5. Statistical analysis

Depending on whether the patients passed away or survived, we divided them into two groups, displayed the factors, and compared the results. To ascertain if RDW levels were related to all-cause mortality, we ran correlation analyses after disclosing and excluding confounders for these independent risk factors.

Categorical variables are presented as counts and percentages, while continuous variables are reported as mean standard deviation (SD) (Gaussian distribution) or median (range) (skewed distribution). To identify differences between various RDW, the *χ*^2^ test (for categorical variables), one-way ANOVA test (for normal distribution), or Kruskal-Wallis H test (for skewed distribution) were used (quartiles). Three different models were created using univariable and multivariable Cox proportional hazards regression models to examine the relationship between RDW and all-cause mortality, including unadjusted models (no adjustment for covariates), minimally adjusted models (adjusting for sociodemographic variables only), and fully adjusted models (adjusting for covariates in Table 1) (16). Effect sizes were recorded along with 95% confidence intervals. Smoothed curve fitting were used to address the non-linearity between RDW and all-cause mortality because approaches based on Cox proportional hazards regression models are frequently accused of failing to handle non-linear models (penalized spline method). We used Cox proportional risk analysis, smoothed curve fitting, subgroup analysis, and Kaplan–Meier survival curves for short, intermediate, and long-term all-cause mortality in unselected critically ill patients.

**Table 1:** Participant’s baseline characteristics (*N* = 26,818).

RDW (%) groups	Total	G1 (<14)	G2 (14–15.49)	G3 (15.5–16.99)	G4 (≥17)	P-value
Number, n	26,818	10,654	7,906	4,282	3,976	
Age (years)	74.2 ± 54.3	67.1 ± 47.8	81.4 ± 61.0	80.0 ± 58.0	73.0 ± 49.6	<0.001
Gender, n (%)						<0.001
Male	15,149 (56.5%)	6,515 (61.2%)	4,284 (54.2%)	2,220 (51.8%)	2,130 (53.6%)	
Female	11,669 (43.5%)	4,139 (38.8%)	3,622 (45.8%)	2062 (48.2%)	1846 (46.4%)	
Admission type, n (%)						<0.001
Emergency	20,749 (77.4%)	8,167 (76.7%)	5,930 (75.0%)	3,367 (78.6%)	3,285 (82.6%)	
Elective	5,371 (20.0%)	2,178 (20.4%)	1772 (22.4%)	816 (19.1%)	605 (15.2%)	
Urgent	698 (2.6%)	309 (2.9%)	204 (2.6%)	99 (2.3%)	86 (2.2%)	
Insurance, n (%)						<0.001
Private	8,733 (32.6%)	4,634 (43.5%)	2,114 (26.7%)	979 (22.9%)	1,006 (25.3%)	
Medicaid	2,148 (8.0%)	820 (7.7%)	585 (7.4%)	358 (8.4%)	385 (9.7%)	
Medicare	14,927 (55.7%)	4,591 (43.1%)	4,997 (63.2%)	2,863 (66.9%)	2,476 (62.3%)	
Government	668 (2.5%)	368 (3.5%)	147 (1.9%)	62 (1.4%)	91 (2.3%)	
Self Pay	342 (1.3%)	241 (2.3%)	63 (0.8%)	20 (0.5%)	18 (0.5%)	
Vital signs						
Heart rate (bpm)	85.5 ± 15.6	84.3 ± 15.3	85.7 ± 15.3	86.4 ± 15.9	87.8 ± 16.2	<0.001
SBP (mmHg)	119.1 ± 17.3	119.9 ± 16.3	119.4 ± 17.2	118.8 ± 17.8	116.9 ± 19.1	<0.001
DBP (mmHg)	59.3 ± 10.7	60.5 ± 10.0	58.9 ± 10.5	58.3 ± 10.9	58.2 ± 12.1	<0.001
respiratory rate (bpm)	18.7 ± 4.1	18.2 ± 3.8	18.8 ± 4.1	19.2 ± 4.2	19.5 ± 4.4	<0.001
Temperature (°C)	36.9 ± 0.6	37.0 ± 0.6	36.9 ± 0.6	36.8 ± 0.6	36.7 ± 0.7	<0.001
SPO2 (%)	97.3 ± 2.8	97.5 ± 2.5	97.3 ± 2.8	97.2 ± 2.6	97.0 ± 3.5	<0.001
Laboratory parameters						
Anion gap (mmol/L)	14.4 ± 3.6	13.9 ± 3.1	14.3 ± 3.5	14.8 ± 3.8	15.7 ± 4.3	<0.001
Bicarbonate (mmol/L)	23.7 ± 4.5	24.0 ± 3.7	23.7 ± 4.5	23.7 ± 5.2	23.2 ± 5.3	<0.001
Creatinine (mEq/L)	1.4 ± 1.5	1.0 ± 0.8	1.4 ± 1.4	1.8 ± 2.0	2.3 ± 2.2	<0.001
Glucose (mg/dL)	144.9 ± 55.0	146.9 ± 56.6	147.3 ± 54.4	142.9 ± 53.2	136.7 ± 52.5	<0.001
Hematocrit (%)	32.6 ± 5.2	34.6 ± 5.0	32.2 ± 5.0	30.8 ± 4.6	30.0 ± 5.0	<0.001
Hemoglobin (g/dL)	11.0 ± 1.9	11.9 ± 1.8	10.9 ± 1.7	10.2 ± 1.6	9.8 ± 1.6	<0.001
Platelet (10^9^/L)	223.7 ± 111.8	224.9 ± 87.1	222.0 ± 111.2	228.5 ± 130.2	218.5 ± 145.1	<0.001
Potassium (mmol/L)	4.2 ± 0.6	4.1 ± 0.5	4.2 ± 0.5	4.3 ± 0.6	4.3 ± 0.7	<0.001
Sodium(mmol/L)	138.5 ± 4.3	138.6 ± 3.8	138.6 ± 4.2	138.5 ± 4.6	138.1 ± 5.0	<0.001
Bun (mg/dL)	25.8 ± 21.1	18.5 ± 13.5	25.8 ± 19.8	32.6 ± 24.0	37.8 ± 27.7	<0.001
WBC (10^9^/L)	12.3 ± 8.2	12.2 ± 5.0	12.2 ± 6.7	12.3 ± 8.6	12.6 ± 14.9	0.047
RDW (%)	14.9 ± 2.1	13.2 ± 0.5	14.6 ± 0.4	16.2 ± 0.4	18.9 ± 1.8	<0.001
RBC (10^12^/L)	3.7 ± 0.6	3.8 ± 0.6	3.6 ± 0.6	3.5 ± 0.6	3.4 ± 0.7	<0.001
Scoring systems						
SOFA	4.1 ± 3.0	3.1 ± 2.4	4.2 ± 2.9	4.7 ± 3.1	5.6 ± 3.5	<0.001
SAPSII	34.5 ± 14.2	29.6 ± 12.9	35.8 ± 13.5	38.3 ± 14.0	40.5 ± 14.9	<0.001
EVCI	5.2 ± 6.7	2.6 ± 5.1	5.1 ± 6.3	7.7 ± 7.1	9.5 ± 7.5	<0.001
Comorbidities, n (%)						
Congestive heart failure	4,643 (17.3%)	805 (7.6%)	1,443 (18.3%)	1,197 (28.0%)	1,198 (30.1%)	<0.001
Cardiac arrhythmias	4,406 (16.4%)	1,015 (9.5%)	1,425 (18.0%)	1,034 (24.1%)	932 (23.4%)	<0.001
Valvular disease	1,570 (5.9%)	352 (3.3%)	487 (6.2%)	398 (9.3%)	333 (8.4%)	<0.001
Pulmonary circulation	755 (2.8%)	165 (1.5%)	227 (2.9%)	184 (4.3%)	179 (4.5%)	<0.001
Peripheral vascular	2093 (7.8%)	608 (5.7%)	724 (9.2%)	402 (9.4%)	359 (9.0%)	<0.001
Hypertension	2,399 (8.9%)	289 (2.7%)	658 (8.3%)	669 (15.6%)	783 (19.7%)	<0.001
Chronic pulmonary	4,729 (17.6%)	1,395 (13.1%)	1,581 (20.0%)	980 (22.9%)	773 (19.4%)	<0.001
Diabetes uncomplicated	5,180 (19.3%)	1,657 (15.6%)	1746 (22.1%)	992 (23.2%)	785 (19.7%)	<0.001
Diabetes complicated	1,684 (6.3%)	316 (3.0%)	505 (6.4%)	405 (9.5%)	458 (11.5%)	<0.001
Hypothyroidism	2,302 (8.6%)	658 (6.2%)	734 (9.3%)	491 (11.5%)	419 (10.5%)	<0.001
Renal failure	3,203 (11.9%)	348 (3.3%)	851 (10.8%)	904 (21.1%)	1,100 (27.7%)	<0.001
Liver disease	1,425 (5.3%)	217 (2.0%)	333 (4.2%)	360 (8.4%)	515 (13.0%)	<0.001
Coagulopathy	2,585 (9.6%)	441 (4.1%)	734 (9.3%)	573 (13.4%)	837 (21.1%)	<0.001
Blood loss anemia	631 (2.4%)	101 (0.9%)	178 (2.3%)	162 (3.8%)	190 (4.8%)	<0.001
Deficiency anemias	4,169 (15.5%)	1,057 (9.9%)	1,220 (15.4%)	900 (21.0%)	992 (24.9%)	<0.001
30-day mortality	3,755 (14.0%)	849 (8.0%)	1,017 (12.9%)	816 (19.1%)	1,073 (27.0%)	<0.001
90-day mortality	5,209 (19.4%)	1,081 (10.1%)	1,412 (17.9%)	1,194 (27.9%)	1,522 (38.3%)	<0.001
365-day mortality	7,587 (28.3%)	1,531 (14.4%)	2,144 (27.1%)	1734 (40.5%)	2,178 (54.8%)	<0.001
4-year mortality	11,385 (42.5%)	2,531 (23.8%)	3,426 (43.3%)	2,558 (59.7%)	2,870 (72.2%)	<0.001

SBP, Systolic blood pressure; RBC, red blood cell; PTT, partial thromboplastin time; BUN, blood urea nitrogen; WBC, white blood cell; PT, prothrombin time; RDW, Red Blood Cell Distribution Width; DBP, Diastolic blood pressure; SAPSII, simplified acute physiology score II; EVCI, Elixhauser-van Walraven Comorbidity Index, SOFA sequential organ failure assessment.

If non-linearity was found, we first used a recursive technique to determine the inflection point, after which we built Cox proportional hazards regression models on either side of the inflection point. The statistical software packages R (R Foundation)^1^ and EmpowerStats (^2^X&Y Solutions, Inc., Boston, MA) were used for all analyses. Statistical significance was defined as a two-sided *p* value less than 0.05 (17).

## 3. Results

### 3.1. Baseline characteristics

A total of 26,818 patients were includedin the fully adjusted model in our study for the final data analysis after screening in accordance with acceptable conditions; 56.5% of them were male and 43.5% were female, with a mean age of 74.2 ± 54.3 years. It objectively displays baseline features of these chosen participants, such as population characteristics, vital signs, laboratory values, physiological scores, and co-morbidities stated in Table 1. Flowchart showing the process we used to choose studies. The findings illustrated statistically significant difference in all indicators across the distinct RDW (%) groups (all *p* values<0.05). Participants with the highest group of RDW(mmol/L) (RDW ≥ 17%) showed lower values for SBP, DBP, Temperature, SPO2, bicarbonate, glucose, hematocrit, hemoglobin, sodium, and RBC, and higher values for heart rate, respiratory rate, anion gap, creatinine, potassium, Bun, WBC, phosphate, SOFA, SAPSII, and EVCI. Congestive heart failure, pulmonary circulation, hypertension, complex diabetes, renal failure, liver illness, coagulopathy, blood loss anemia, deficiency, and anemias were more common in this group of individuals in contrast with those in the other subgroups (see Figure 1).

### 3.2. Results of the adjusted and unadjusted cox proportional hazard models

We used three models to evaluate the independent connection of RDW levels on all-cause mortality in unselected critically ill patients. The results are shown in Table 2 as effect sizes (risk ratio HRs) and 95 percent confidence intervals.

**Table 2:** Association of RDW with mortality.

Variable	Crude model HR (95% CIs) *p*-value	Model I HR (95% CIs) *p*-value	Model II HR (95% CIs) *p*-value
*30-day mortality, n (%)*			
RDW (%)	1.20 (1.19, 1.22) <0.0001	1.21 (1.19, 1.22) <0.0001	1.10 (1.08, 1.12) <0.0001
*RDW (%) groups*			
<14	Ref	Ref	Ref
> = 14, <15.5	1.65 (1.51, 1.81) <0.0001	1.53 (1.39, 1.67) <0.0001	1.14 (1.04, 1.26) 0.0075
> = 15.5, <17	2.51 (2.28, 2.77) <0.0001	2.34 (2.13, 2.58) <0.0001	1.46 (1.31, 1.63) <0.0001
> = 17	3.73 (3.41, 4.08) <0.0001	3.64 (3.33, 3.98) <0.0001	1.85 (1.66, 2.07) <0.0001
*90-day mortality, n (%)*			
RDW (%)	1.22 (1.21, 1.24) <0.0001	1.23 (1.22, 1.24) <0.0001	1.11 (1.10, 1.13) <0.0001
*RDW (%) groups*			
<14	Ref	Ref	Ref
> = 14, <15.5	1.83 (1.69, 1.98) <0.0001	1.69 (1.56, 1.83) <0.0001	1.24 (1.14, 1.35) <0.0001
> = 15.5, <17	2.99 (2.75, 3.25) <0.0001	2.80 (2.58, 3.04) <0.0001	1.70 (1.54, 1.86) <0.0001
> = 17	4.40 (4.07, 4.76) <0.0001	4.31 (3.99, 4.66) <0.0001	2.12 (1.93, 2.34) <0.0001
*365-day mortality, n (%)*			
RDW (%)	1.24 (1.23, 1.24) <0.0001	1.24 (1.23, 1.25) <0.0001	1.13 (1.12, 1.14) <0.0001
*RDW (%) groups*			
<14	Ref	Ref	Ref
> = 14, <15.5	2.02 (1.89, 2.16) <0.0001	1.87 (1.75, 2.00) <0.0001	1.38 (1.29, 1.48) <0.0001
> = 15.5, <17	3.28 (3.06, 3.51) <0.0001	3.08 (2.88, 3.30) <0.0001	1.86 (1.72, 2.01) <0.0001
> = 17	4.96 (4.65, 5.30) <0.0001	4.87 (4.56, 5.20) <0.0001	2.42 (2.23, 2.62) <0.0001
*4-year mortality, n (%)*			
RDW (%)	1.23 (1.23, 1.24) <0.0001	1.24 (1.23, 1.25) <0.0001	1.13 (1.12, 1.14) <0.0001
*RDW (%) groups*			
<14	Ref	Ref	Ref
> = 14, <15.5	2.07 (1.96, 2.18) <0.0001	1.92 (1.82, 2.02) <0.0001	1.44 (1.36, 1.52) <0.0001
> = 15.5, <17	3.31 (3.13, 3.50) <0.0001	3.12 (2.95, 3.29) <0.0001	1.93 (1.82, 2.06) <0.0001
> = 17	4.76 (4.52, 5.03) <0.0001	4.71 (4.46, 4.97) <0.0001	2.47 (2.31, 2.63) <0.0001

RDW, Red Blood Cell Distribution Width; HR, Hazard Ratio.

The unadjusted model’s HRs for 30-day all-cause mortality had a value of 1.20 [1.20, 95% CIs (1.19, 1.22)], implying a 20% increased risk of 30-day all-cause mortality, *ceteris paribus*, a 22% increased risk of 90-day all-cause mortality, an 24% increased risk of 365-day all-cause mortality, and an 23% increased risk of 4-year all-cause mortality.

Model 1 (minimally-adjusted model) was defined by the relationship between RDW levels and mortality risk in the minimally-adjusted model, where the HRs for 30-day all-cause mortality were 1.21 [1.21, 95%CIs (1.19, 1.22)], implying a 21% increase in the risk of 30-day all-cause mortality, *ceteris paribus*, a 23% increase in the risk of 90-day all-cause mortality, a 24% increase in the risk of 365-day all-cause mortality, and 24% increase in the risk of 4-years all-cause mortality.

Model 2 (fully adjusted mode) is characterized by the association between the RDW levels linked with mortality risk in the fully adjusted model. The HRs for 30-day all-cause mortality are 1.10 [1.10, 95%CIs (1.08, 1.12)], reflecting a 10% increase in risk for 30-day all-cause mortality. The risk of 90-day all-cause mortality is increased by 11%, the risk of 365-day all-cause mortality is increased by 13%, and the risk of 4-year all-cause mortality is increased by 13% as a result.

For mortality at 30 days, 90 days, 365 days, and 4 years, we performed sensitivity analyses, treating RDW levels by categorical variables into four groups. We discovered that the results were consistent with RDW as a continuous variable, and all *p*-values were less than 0.05, making the differences statistically significant.

### 3.3. Subgroup analysis

We used age (years), gender, admission type, insurance type, heart rate (bpm), SBP (mmHg), DBP (mmHg), respiratory rate (bpm), temperature (°C), SPO2 (%), anion gap (mmol/L), bicarbonate level (mmol/L), creatinine level (mEq/L), glucose level (mg/dL), hematocrit level (%), hemoglobin level (g/dL), platelet level (10^9^/L), potassium level (mmol/L), sodium level (mmol/L), Bun level (mg/dL), WBC count (10^9^/L), RBC count (10^12^/L), SOFA score, SAPS II score, EVCI score, congestive heart failure, cardiac arrhythmias, blood loss anemia, valvular disease, pulmonary circulation, peripheral vascular disease, hypertension, chronic pulmonary disease, uncomplicated diabetes, complicated diabetes, hypothyroidism, renal failure, liver disease, coagulopathy, blood loss anemia, and deficiency anemias as the stratification parameters to examine the patterns of their effect sizes (Table 3). When compared across subgroups and overall, our results are quite trustworthy and consistent. Each subgroup’s results from the stratified analysis point in the same direction, with statistically significant effect values and a 95 percent confidence interval.

**Table 3:** Each subgroup’s RWD effect size on mortality in prespecified and exploratory subgroups.

Characteristic		30-day mortality, *n* (%)		90-day mortality, *n* (%)		365-day mortality, *n* (%)		4-year mortality, *n* (%)	
	*N*	HR (95% CI)	P for interaction	HR (95% CI)	P for interaction	HR (95% CI)	P for interaction	HR (95% CI)	P for interaction
Age (years) groups			<0.0001		<0.0001		<0.0001		<0.0001
<60	10,490	1.27 (1.25, 1.30)		1.30 (1.28, 1.32)		1.30 (1.29, 1.32)		1.29 (1.28, 1.31)	
> = 60, <80	11,057	1.20 (1.18, 1.22)		1.22 (1.20, 1.24)		1.23 (1.22, 1.25)		1.23 (1.22, 1.24)	
> = 80	5,271	1.12 (1.10, 1.15)		1.14 (1.12, 1.17)		1.16 (1.14, 1.18)		1.15 (1.13, 1.17)	
Gender, n (%)			0.0067		0.0006		<0.0001		<0.0001
Male	15,149	1.22 (1.20, 1.24)		1.24 (1.23, 1.26)		1.25 (1.24, 1.27)		1.25 (1.24, 1.26)	
Female	11,669	1.18 (1.16, 1.20)		1.20 (1.18, 1.22)		1.21 (1.19, 1.22)		1.20 (1.19, 1.22)	
Admission type, n (%)			<0.0001		<0.0001		<0.0001		<0.0001
Emergency	20,749	1.19 (1.17, 1.20)		1.21 (1.20, 1.22)		1.22 (1.21, 1.23)		1.22 (1.21, 1.23)	
Elective	5,371	1.30 (1.25, 1.35)		1.32 (1.28, 1.36)		1.30 (1.27, 1.33)		1.28 (1.26, 1.30)	
Urgent	698	1.29 (1.20, 1.38)		1.27 (1.19, 1.35)		1.30 (1.23, 1.38)		1.28 (1.22, 1.34)	
Insurance, n (%)			<0.0001		<0.0001		<0.0001		<0.0001
Private	8,733	1.27 (1.24, 1.29)		1.29 (1.27, 1.31)		1.29 (1.28, 1.31)		1.29 (1.27, 1.30)	
Medicaid	2,148	1.22 (1.17, 1.28)		1.22 (1.18, 1.27)		1.24 (1.20, 1.28)		1.24 (1.21, 1.27)	
Medicare	14,927	1.15 (1.14, 1.17)		1.17 (1.16, 1.19)		1.18 (1.17, 1.20)		1.18 (1.17, 1.19)	
Government	668	1.38 (1.28, 1.48)		1.40 (1.31, 1.50)		1.39 (1.31, 1.47)		1.35 (1.28, 1.42)	
Self Pay	342	1.26 (1.10, 1.43)		1.26 (1.11, 1.43)		1.26 (1.11, 1.43)		1.29 (1.15, 1.44)	
Heart rate (bpm) groups			0.5383		0.5341		0.4751		0.2323
<60	959	1.19 (1.11, 1.28)		1.24 (1.17, 1.32)		1.27 (1.21, 1.33)		1.29 (1.24, 1.34)	
> = 60, <90	15,916	1.21 (1.19, 1.23)		1.23 (1.21, 1.24)		1.24 (1.23, 1.25)		1.24 (1.23, 1.25)	
> = 90	9,562	1.19 (1.17, 1.21)		1.21 (1.20, 1.23)		1.22 (1.21, 1.24)		1.22 (1.21, 1.24)	
SBP (mmHg) groups			<0.0001		<0.0001		<0.0001		<0.0001
<90	489	1.07 (1.02, 1.12)		1.08 (1.04, 1.13)		1.09 (1.05, 1.14)		1.09 (1.05, 1.14)	
> = 90, <140	22,647	1.23 (1.21, 1.24)		1.25 (1.23, 1.26)		1.25 (1.24, 1.26)		1.25 (1.24, 1.26)	
> = 140	3,299	0.99 (0.95, 1.04)		1.04 (1.00, 1.08)		1.11 (1.08, 1.14)		1.13 (1.11, 1.16)	
DBP (mmHg) groups			<0.0001		0.0003		0.0011		0.1194
<60	14,924	1.22 (1.20, 1.24)		1.23 (1.22, 1.25)		1.24 (1.23, 1.25)		1.24 (1.23, 1.25)	
> = 60, <90	11,277	1.17 (1.15, 1.20)		1.20 (1.18, 1.22)		1.23 (1.21, 1.24)		1.23 (1.21, 1.24)	
> = 90	234	0.85 (0.69, 1.06)		0.94 (0.78, 1.12)		1.00 (0.88, 1.14)		1.15 (1.06, 1.25)	
Respiratory rate (bpm) groups			0.0005		<0.0001		<0.0001		<0.0001
<12	381	1.10 (0.97, 1.24)		1.15 (1.05, 1.27)		1.16 (1.07, 1.26)		1.16 (1.08, 1.25)	
> = 12, <20	17,640	1.22 (1.20, 1.24)		1.24 (1.23, 1.26)		1.25 (1.24, 1.27)		1.25 (1.24, 1.26)	
> = 20	8,363	1.17 (1.15, 1.18)		1.18 (1.17, 1.20)		1.19 (1.18, 1.21)		1.19 (1.18, 1.20)	
Temperature (°C) groups			0.0054		0.0405		0.0319		0.0462
<36.3	4,019	1.19 (1.17, 1.22)		1.20 (1.18, 1.23)		1.21 (1.19, 1.23)		1.20 (1.19, 1.22)	
> = 36.3, <37.2	14,953	1.21 (1.19, 1.23)		1.23 (1.22, 1.25)		1.24 (1.23, 1.26)		1.24 (1.23, 1.25)	
> = 37.2	7,386	1.16 (1.13, 1.18)		1.19 (1.17, 1.22)		1.21 (1.19, 1.23)		1.21 (1.20, 1.23)	
SPO2 (%) groups			0.0741		0.0077		0.0023		0.0003
<95	2,576	1.17 (1.14, 1.20)		1.18 (1.15, 1.21)		1.19 (1.16, 1.21)		1.18 (1.16, 1.20)	
> = 95	23,833	1.21 (1.19, 1.22)		1.23 (1.22, 1.24)		1.24 (1.23, 1.25)		1.24 (1.23, 1.25)	
Anion gap (mmol/L) groups			<0.0001		<0.0001		<0.0001		<0.0001
<8	185	1.32 (1.12, 1.56)		1.29 (1.13, 1.47)		1.30 (1.18, 1.44)		1.35 (1.24, 1.47)	
> = 8, <16	18,276	1.21 (1.19, 1.23)		1.24 (1.22, 1.25)		1.25 (1.23, 1.26)		1.24 (1.23, 1.25)	
> = 16	7,557	1.15 (1.13, 1.17)		1.16 (1.15, 1.18)		1.18 (1.17, 1.20)		1.18 (1.17, 1.20)	
Bicarbonate (mmol/L) groups			0.0004		0.0002		<0.0001		<0.0001
<22	7,676	1.18 (1.16, 1.20)		1.20 (1.18, 1.22)		1.21 (1.19, 1.22)		1.21 (1.19, 1.22)	
> = 22, <27	13,606	1.22 (1.20, 1.25)		1.24 (1.22, 1.26)		1.25 (1.24, 1.27)		1.25 (1.24, 1.26)	
> = 27	5,362	1.15 (1.12, 1.18)		1.18 (1.16, 1.21)		1.20 (1.18, 1.22)		1.20 (1.18, 1.21)	
Creatinine (mEq/L) groups			<0.0001		<0.0001		<0.0001		<0.0001
<0.5	969	1.18 (1.10, 1.26)		1.21 (1.14, 1.27)		1.23 (1.18, 1.29)		1.21 (1.17, 1.26)	
> = 0.5, <1.2	16,772	1.22 (1.20, 1.24)		1.24 (1.23, 1.26)		1.25 (1.24, 1.26)		1.25 (1.23, 1.26)	
> = 1.2	9,027	1.13 (1.11, 1.15)		1.15 (1.13, 1.16)		1.16 (1.14, 1.17)		1.16 (1.14, 1.17)	
Glucose (mg/dL) groups			0.0003		0.0323		0.9333		0.3475
<70	192	1.18 (1.08, 1.28)		1.25 (1.16, 1.34)		1.24 (1.16, 1.32)		1.24 (1.17, 1.32)	
> = 70, <110	5,562	1.26 (1.23, 1.29)		1.25 (1.23, 1.28)		1.24 (1.22, 1.26)		1.23 (1.21, 1.24)	
> = 110	21,013	1.19 (1.17, 1.21)		1.22 (1.20, 1.23)		1.23 (1.22, 1.24)		1.23 (1.22, 1.24)	
Hematocrit (%) groups			0.0494		0.0167		0.0013		<0.0001
<37	21,397	1.20 (1.19, 1.22)		1.22 (1.21, 1.23)		1.23 (1.22, 1.24)		1.22 (1.21, 1.23)	
> = 37, <50	5,369	1.25 (1.21, 1.29)		1.27 (1.23, 1.30)		1.28 (1.25, 1.31)		1.29 (1.27, 1.32)	
> = 50	52	1.12 (0.91, 1.40)		1.15 (0.94, 1.41)		1.16 (0.97, 1.39)		1.21 (1.04, 1.40)	
Hemoglobin (g/dL) groups			<0.0001		<0.0001		<0.0001		<0.0001
<11	14,359	1.19 (1.17, 1.20)		1.20 (1.19, 1.21)		1.20 (1.19, 1.22)		1.20 (1.19, 1.21)	
> = 11, <16.5	12,355	1.26 (1.24, 1.29)		1.28 (1.26, 1.31)		1.30 (1.28, 1.32)		1.31 (1.29, 1.33)	
> = 16.5	103	1.13 (0.94, 1.35)		1.16 (0.98, 1.36)		1.18 (1.02, 1.36)		1.23 (1.09, 1.39)	
Platelet (10^9^/L) groups			<0.0001		<0.0001		<0.0001		<0.0001
<100	2,174	1.15 (1.12, 1.18)		1.15 (1.13, 1.18)		1.15 (1.13, 1.17)		1.14 (1.12, 1.16)	
> = 100, <300	19,717	1.22 (1.20, 1.24)		1.25 (1.23, 1.26)		1.27 (1.25, 1.28)		1.27 (1.26, 1.28)	
> = 300	4,921	1.13 (1.10, 1.16)		1.15 (1.13, 1.18)		1.17 (1.15, 1.19)		1.17 (1.15, 1.19)	
Potassium (mmol/L) groups			0.0049		<0.0001		<0.0001		<0.0001
<3.5	1884	1.17 (1.12, 1.21)		1.19 (1.15, 1.23)		1.21 (1.18, 1.24)		1.21 (1.18, 1.24)	
> = 3.5, <5.5	24,165	1.21 (1.19, 1.22)		1.23 (1.22, 1.24)		1.24 (1.23, 1.25)		1.24 (1.23, 1.25)	
> = 5.5	744	1.11 (1.05, 1.17)		1.10 (1.05, 1.16)		1.11 (1.06, 1.15)		1.11 (1.07, 1.14)	
Sodium(mmol/L) groups			0.0218		0.0004		<0.0001		<0.0001
<135	3,898	1.20 (1.17, 1.22)		1.20 (1.18, 1.22)		1.20 (1.18, 1.22)		1.18 (1.16, 1.19)	
> = 135, <145	21,500	1.20 (1.18, 1.21)		1.23 (1.21, 1.24)		1.24 (1.23, 1.25)		1.25 (1.24, 1.25)	
> = 145	1,374	1.13 (1.09, 1.17)		1.14 (1.11, 1.18)		1.15 (1.12, 1.18)		1.16 (1.13, 1.19)	
Bun (mg/dL) groups			0.0004		<0.0001		<0.0001		<0.0001
<9	2,113	1.21 (1.14, 1.28)		1.24 (1.18, 1.30)		1.25 (1.21, 1.30)		1.24 (1.21, 1.28)	
> = 9, <20	12,185	1.20 (1.17, 1.24)		1.25 (1.23, 1.27)		1.27 (1.25, 1.29)		1.27 (1.25, 1.28)	
> = 20	12,466	1.14 (1.12, 1.16)		1.15 (1.14, 1.17)		1.16 (1.15, 1.17)		1.16 (1.15, 1.17)	
WBC (10^9^/L) groups			0.0049		<0.0001		<0.0001		<0.0001
<4	843	1.12 (1.07, 1.18)		1.12 (1.07, 1.16)		1.12 (1.09, 1.16)		1.11 (1.08, 1.15)	
> = 4, <10	9,932	1.21 (1.18, 1.23)		1.23 (1.21, 1.25)		1.23 (1.22, 1.25)		1.23 (1.22, 1.24)	
> = 10	16,040	1.21 (1.20, 1.23)		1.23 (1.22, 1.25)		1.25 (1.23, 1.26)		1.24 (1.23, 1.26)	
RBC (10^12^/L) groups			0.236		0.0373		<0.0001		<0.0001
<3.5	11,796	1.20 (1.18, 1.22)		1.21 (1.20, 1.23)		1.22 (1.20, 1.23)		1.21 (1.20, 1.22)	
> = 3.5, <5.5	14,870	1.22 (1.20, 1.24)		1.24 (1.22, 1.26)		1.26 (1.24, 1.28)		1.26 (1.25, 1.27)	
> = 5.5	150	1.17 (1.04, 1.31)		1.16 (1.04, 1.30)		1.19 (1.09, 1.30)		1.21 (1.13, 1.30)	
SOFA groups			0.0103		<0.0001		<0.0001		<0.0001
<5	16,904	1.19 (1.16, 1.21)		1.23 (1.21, 1.24)		1.25 (1.23, 1.26)		1.25 (1.24, 1.26)	
> = 5	9,914	1.14 (1.13, 1.16)		1.16 (1.15, 1.18)		1.17 (1.16, 1.19)		1.18 (1.17, 1.19)	
SAPSII groups			0.0001		<0.0001		<0.0001		<0.0001
<39	17,631	1.19 (1.16, 1.21)		1.24 (1.22, 1.26)		1.25 (1.24, 1.27)		1.25 (1.24, 1.27)	
> = 39	9,187	1.12 (1.11, 1.14)		1.14 (1.12, 1.15)		1.15 (1.14, 1.16)		1.14 (1.13, 1.15)	
EVCI groups			<0.0001		<0.0001		<0.0001		<0.0001
<8	18,570	1.20 (1.18, 1.22)		1.22 (1.20, 1.24)		1.24 (1.22, 1.25)		1.24 (1.23, 1.25)	
> = 8	8,248	1.13 (1.11, 1.14)		1.14 (1.12, 1.15)		1.14 (1.13, 1.15)		1.13 (1.12, 1.14)	
Congestive heart failure			<0.0001		<0.0001		<0.0001		<0.0001
No	22,175	1.22 (1.20, 1.23)		1.24 (1.22, 1.25)		1.25 (1.24, 1.26)		1.24 (1.23, 1.25)	
Yes	4,643	1.11 (1.08, 1.14)		1.12 (1.10, 1.15)		1.13 (1.11, 1.15)		1.12 (1.11, 1.14)	
Cardiac arrhythmias			<0.0001		<0.0001		<0.0001		<0.0001
No	22,412	1.22 (1.20, 1.23)		1.24 (1.22, 1.25)		1.24 (1.23, 1.26)		1.24 (1.23, 1.25)	
Yes	4,406	1.11 (1.08, 1.14)		1.13 (1.11, 1.16)		1.15 (1.13, 1.17)		1.15 (1.14, 1.17)	
Valvular disease			0.0164		<0.0001		<0.0001		<0.0001
No	25,248	1.21 (1.19, 1.22)		1.23 (1.22, 1.24)		1.24 (1.23, 1.25)		1.24 (1.23, 1.24)	
Yes	1,570	1.15 (1.11, 1.20)		1.15 (1.12, 1.19)		1.16 (1.13, 1.19)		1.15 (1.12, 1.18)	
Pulmonary circulation			0.0378		0.0057		0.0007		0.0003
No	26,063	1.21 (1.19, 1.22)		1.23 (1.21, 1.24)		1.24 (1.23, 1.25)		1.23 (1.23, 1.24)	
Yes	755	1.14 (1.08, 1.21)		1.15 (1.10, 1.21)		1.16 (1.11, 1.20)		1.16 (1.12, 1.20)	
Peripheral vascular			0.6551		0.3955		0.6589		0.9845
No	24,725	1.21 (1.19, 1.22)		1.23 (1.21, 1.24)		1.24 (1.23, 1.25)		1.23 (1.22, 1.24)	
Yes	2093	1.19 (1.14, 1.25)		1.21 (1.16, 1.25)		1.23 (1.20, 1.27)		1.24 (1.21, 1.27)	
Hypertension			<0.0001		<0.0001		<0.0001		<0.0001
No	24,419	1.22 (1.20, 1.23)		1.23 (1.22, 1.25)		1.24 (1.23, 1.25)		1.24 (1.23, 1.25)	
Yes	2,399	1.10 (1.06, 1.15)		1.12 (1.09, 1.16)		1.13 (1.10, 1.15)		1.13 (1.10, 1.15)	
Chronic pulmonary			0.0043		0.0005		<0.0001		<0.0001
No	22,089	1.21 (1.20, 1.23)		1.23 (1.22, 1.24)		1.24 (1.23, 1.25)		1.24 (1.23, 1.25)	
Yes	4,729	1.16 (1.13, 1.19)		1.18 (1.15, 1.21)		1.19 (1.17, 1.21)		1.19 (1.17, 1.21)	
Diabetes uncomplicated			0.6432		0.2864		0.3093		0.3432
No	21,638	1.21 (1.19, 1.22)		1.23 (1.22, 1.24)		1.24 (1.23, 1.25)		1.23 (1.23, 1.24)	
Yes	5,180	1.20 (1.17, 1.23)		1.21 (1.19, 1.24)		1.23 (1.21, 1.25)		1.23 (1.21, 1.25)	
Diabetes complicated			0.3581		0.3955		0.0861		0.2017
No	25,134	1.21 (1.20, 1.22)		1.23 (1.22, 1.24)		1.24 (1.23, 1.25)		1.23 (1.22, 1.24)	
Yes	1,684	1.18 (1.12, 1.24)		1.21 (1.16, 1.26)		1.21 (1.17, 1.25)		1.23 (1.20, 1.26)	
Hypothyroidism			0.6376		0.8821		0.0968		0.0478
No	24,516	1.21 (1.19, 1.22)		1.23 (1.21, 1.24)		1.24 (1.23, 1.25)		1.23 (1.23, 1.24)	
Yes	2,302	1.20 (1.15, 1.24)		1.22 (1.19, 1.26)		1.21 (1.19, 1.24)		1.21 (1.19, 1.24)	
Renal failure			<0.0001		<0.0001		<0.0001		<0.0001
No	23,615	1.22 (1.20, 1.23)		1.23 (1.22, 1.25)		1.24 (1.23, 1.25)		1.24 (1.23, 1.24)	
Yes	3,203	1.11 (1.08, 1.15)		1.14 (1.11, 1.17)		1.14 (1.12, 1.16)		1.14 (1.12, 1.16)	
Liver disease			0.2201		0.0659		0.0002		<0.0001
No	25,393	1.21 (1.19, 1.22)		1.23 (1.22, 1.24)		1.24 (1.23, 1.25)		1.24 (1.23, 1.25)	
Yes	1,425	1.18 (1.13, 1.23)		1.19 (1.15, 1.23)		1.18 (1.14, 1.21)		1.16 (1.13, 1.19)	
Coagulopathy			<0.0001		<0.0001		<0.0001		<0.0001
No	24,233	1.20 (1.19, 1.22)		1.23 (1.21, 1.24)		1.24 (1.23, 1.25)		1.24 (1.23, 1.25)	
Yes	2,585	1.14 (1.11, 1.17)		1.15 (1.13, 1.17)		1.16 (1.14, 1.18)		1.15 (1.13, 1.17)	
Blood loss anemia			0.1809		0.0063		0.0002		<0.0001
No	26,187	1.21 (1.19, 1.22)		1.23 (1.22, 1.24)		1.24 (1.23, 1.25)		1.24 (1.23, 1.25)	
Yes	631	1.16 (1.08, 1.24)		1.14 (1.08, 1.21)		1.15 (1.10, 1.20)		1.15 (1.11, 1.19)	
Deficiency anemias			0.0074		<0.0001		<0.0001		<0.0001
No	22,649	1.22 (1.21, 1.24)		1.24 (1.23, 1.26)		1.26 (1.24, 1.27)		1.25 (1.24, 1.26)	
Yes	4,169	1.18 (1.14, 1.21)		1.18 (1.15, 1.20)		1.17 (1.15, 1.20)		1.17 (1.15, 1.19)	

Our stratified analysis’ findings for diseases in all major organs demonstrate a high degree of consistency and dependability. In all systems, the patient’s short-, medium-, and long-term mortality is positively correlated with the RDW value: the greater the RDW value, the higher the patient’s short-, medium-, and long-term mortality.

### 3.4. The results of the non-linearity of RWD and all-cause mortality

**Figure 2:**
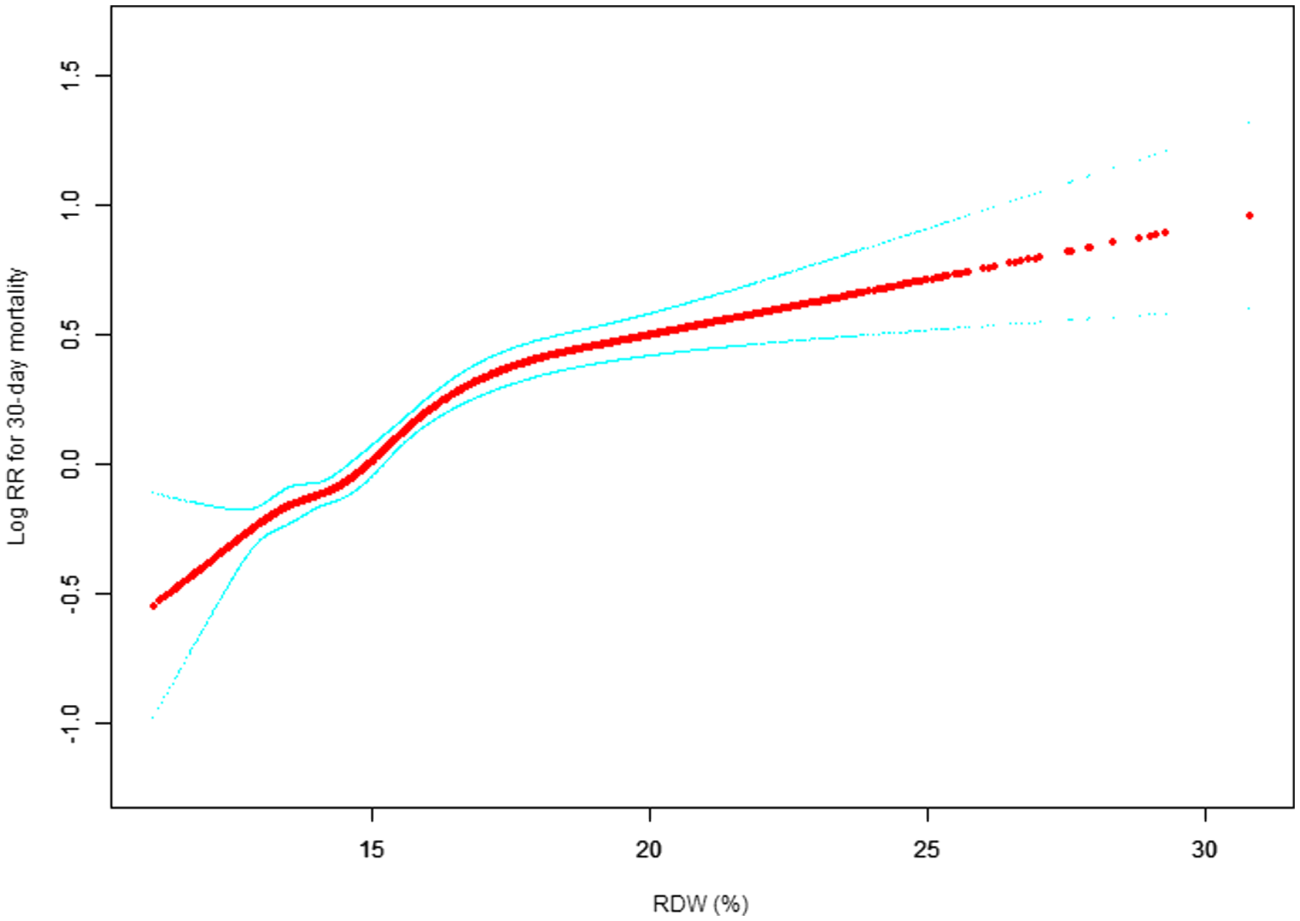
Association between RDW and 30-day all-cause mortality. (After adjustment for other covariates). A generalized additive model (GAM) revealed a threshold, nonlinear relationship between RDW and 30-day mortality. The smooth curve fit between variables is shown by a solid red line. The 95% confidence interval from the fit is represented by imaginary blue line.

**Figure 3:**
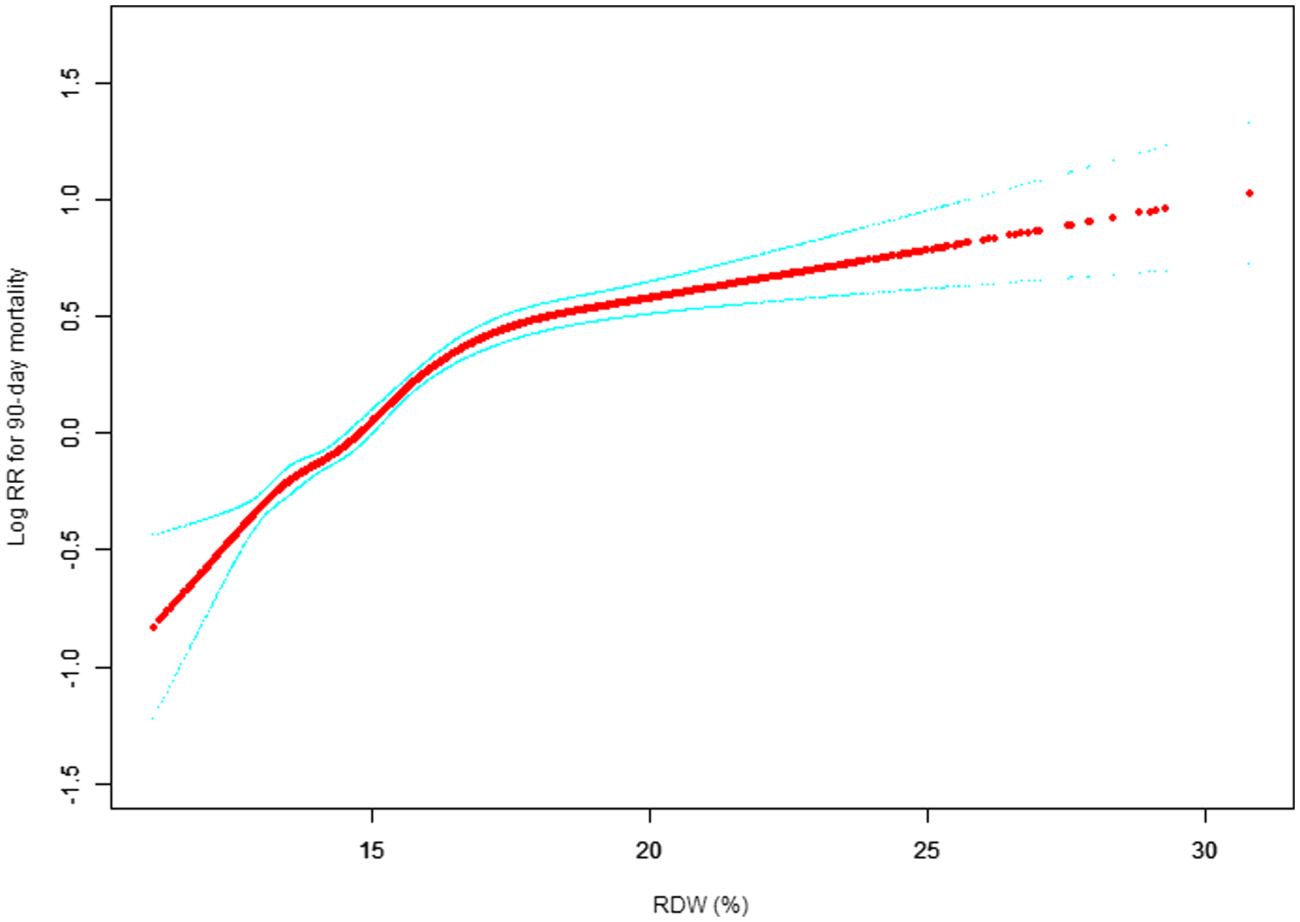
Association between RDW and 90-day all-cause mortality. (After adjustment for other covariates). A generalized additive model (GAM) revealed a threshold, nonlinear relationship between RDW and 30-day mortality. The smooth curve fit between variables is shown by a solid red line. The 95% confidence interval from the fit is represented by imaginary blue line.

**Figure 4:**
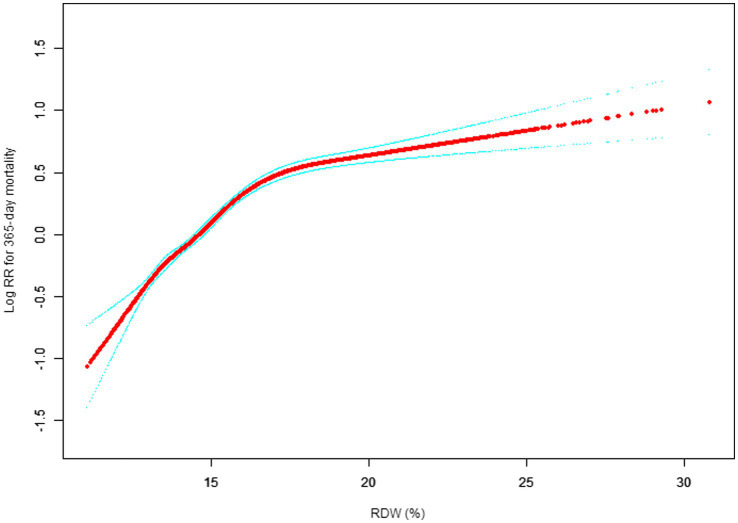
Association between RDW and 365-day all-cause mortality. (After adjustment for other covariates). A generalized additive model (GAM) revealed a threshold, nonlinear relationship between RDW and 30-day mortality. The smooth curve fit between variables is shown by a solid red line. The 95% confidence interval from the fit is represented by imaginary blue line.

**Figure 5:**
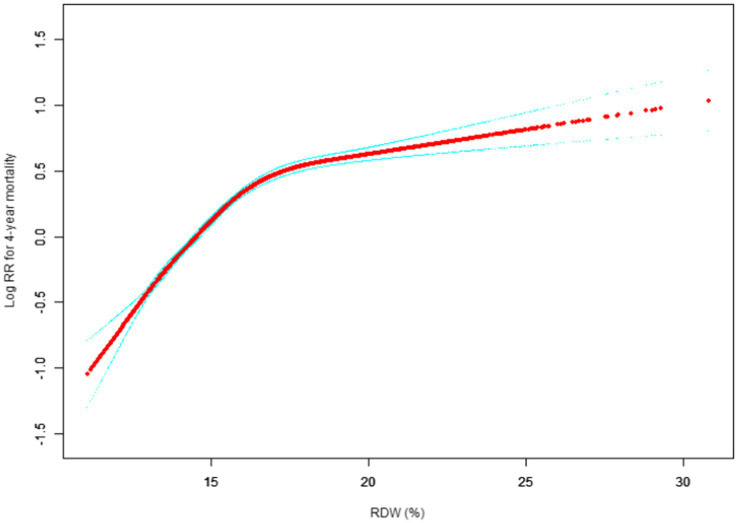
Association between RDW and 4-year all-cause mortality. (After adjustment for other covariates). A generalized additive model (GAM) revealed a threshold, nonlinear relationship between RDW and 30-day mortality. The smooth curve fit between variables is shown by a solid red line. The 95% confidence interval from the fit is represented by imaginary blue line.

Our study investigated the relationship between RDW levels and all-cause mortality in a non-selected population (Figures 2–5) by smoothed curve fit plots and COX proportional risk regression models integrating cubic spline functions reported results about RDW levels showing a non-linear relationship with short-, medium- and long-term all-cause mortality in a non-selected population.

The fully adjusted model showed a non-linear positive correlation, and we adjusted for the following covariaties: age; heart rate; systolic blood pressure (SBP); temperature; pulse oxygen saturation (SPO2); diastolic blood pressure (DBP); respiratory rate; anion gap; albumin level; blood urea nitrogen (Bun) level; Platelet level; sodium level; hemoglobin level; hematocrit level; glucose level; potassium level; creatinine level; bicarbonate level; Phosphate level, Magnesium level, Serum calcium level, the Simplified Acute Physiology Score II (SAPS II); the Sequential Organ Failure Assessment (SOFA) score; and the Elixhauser-van Walraven Comorbidity Index (EVCI); red blood cell (RBC) count; red blood cell distribution width (RDW) level; white blood cells (WBC) count; gender; insurance; admission type; cardiac arrhythmias; valvular disease; pulmonary circulation; congestive heart failure; peripheral vascular; hypertension; chronic pulmonary; diabetes uncomplicated; diabetes complicated; hypothyroidism; renal failure; liver disease; coagulopathy; blood loss anemia; deficiency anemias.

We utilized a Cox proportional risk model and a Cox proportional risk model to fit this association. We chose the best model based on the *p*-values obtained from the log-likelihood ratio test.

### 3.5. Survival status of the patients with different admission RDW levels.

**Figure 6:**
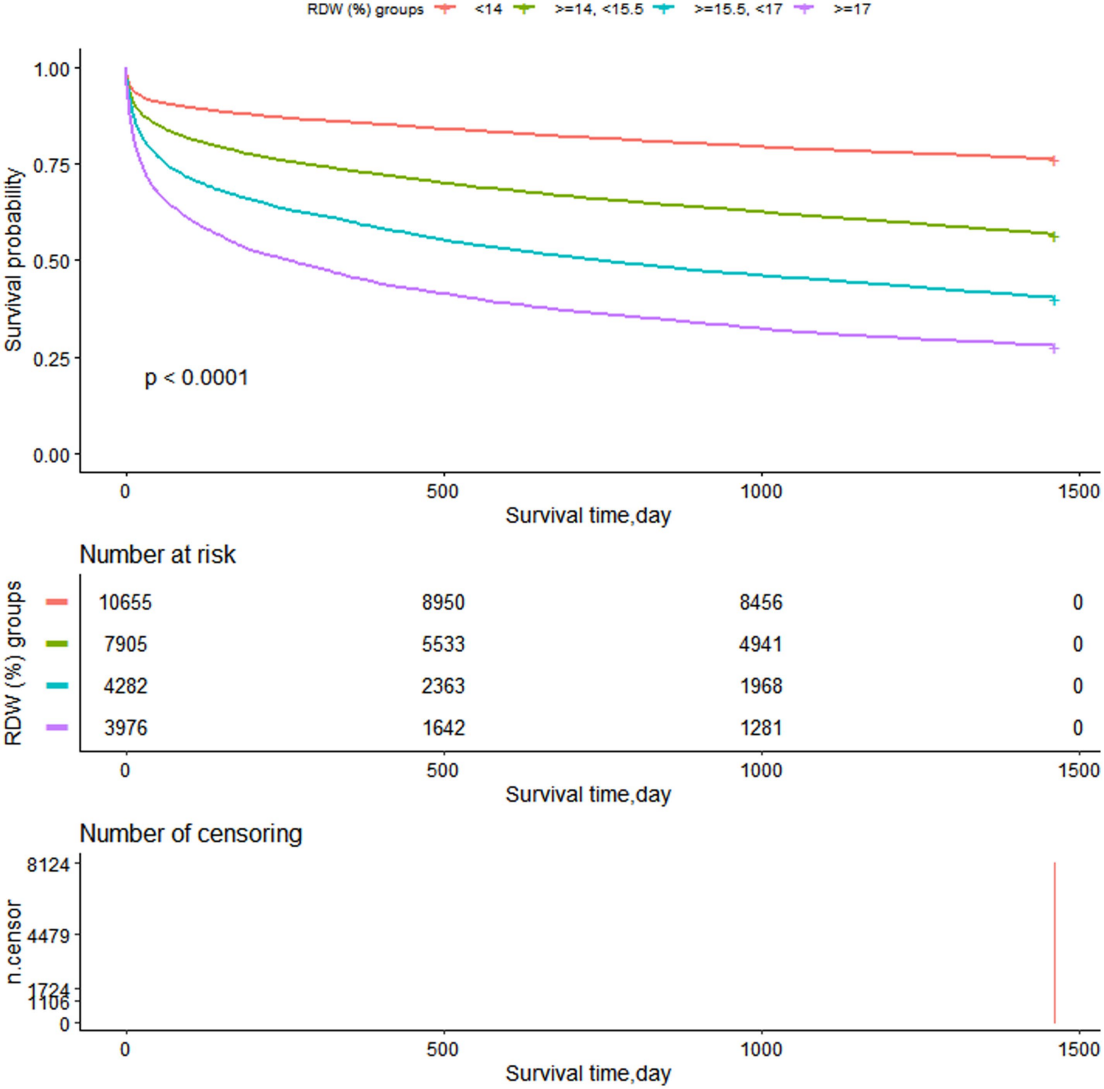
Kaplan–Meier survival curves demonstrating differences in overall survival (years).

Figure 6 illustrates how the K-M survival curves (Figure 6) revealed that patients in each RDW group had survival time values of G1 > G2 > G3 > G4 at any point throughout the 4 years (*p* < 0.0001).

## 4. Discussion

In this study, which is the first to examine the relationship between erythrocyte distribution width levels and short-, medium-, and long-term mortality in unselected critically ill patients, we can see that erythrocyte distribution width levels are positively associated with all-cause mortality in unselected critically ill patients and that increases in 30 days, 90 days, 365 days, and 4-year mortality in unselected critically ill patients are linked to increases in erythrocyte distribute. In our patient population, we observed low RDW groups suggestive of low mortality.

Red blood cell distribution width (RDW), which is typically quantified by a straightforward equation calculated as the standard deviation (SD) of red blood cell volume divided by the mean red blood cell volume (MCV) and multiplied by 100%, is considered to be a measure of the heterogeneity of red blood cell size. One of the standard tests for whole blood cells is erythrocyte distribution width, a metric that indicates the heterogeneity of red blood cell volume that may be determined using a standard blood analyzer. It is a measure of red blood cell size inequality that is objective and is typically stated as RDW-CV or RDW-SD (18), and are frequently used in clinical settings to distinguish between different anemias and small cell hypochromic anemias, such as iron deficiency anemia (19). The investigation into the connection between RDW and unfavorable outcomes began with a 2007 study by Anderson et al. that looked at the relationship between different CBC components and cardiovascular morbidity and death (20). Numerous studies have shown that RDW is linked to mortality outcomes in patients with pulmonary hypertension, patients at risk, general patients, patients without coronary artery disease, and even the general population. RDW is also linked to disease states like heart failure, coronary atherosclerotic heart disease, myocardial infarction, and stroke. RDW is linked to a number of disease endpoint events, including abrupt heart failure in people without heart failure, readmission to the hospital for heart failure, and in-hospital death in patients with myocardial infarction, according to numerous studies (1, 21, 22). RDW has drawn more attention as a regular blood test because of its significance for disease and outcome as well as its low cost and ease of use.

Uncertain pathophysiological mechanisms underlie the association between higher RDW and unfavorable results. Any pathological changes, such as hepatic or renal insufficiency, cardiac insufficiency, inflammatory response, tumor proliferation, nutritional deficiencies, and oxidative stress, can result in elevated RDW (7–11, 23). RDW can be elevated due to red blood cell destruction (such as hemolysis, transfusion), or due to ineffective hematopoiesis. Erythrocytes are said to have a lifespan of 100–130 days on average, according to the literature, though this might vary widely from person to person (24). Erythrocyte density increases and surface area decreases over the course of their lifespan, which may result in a diverse population of erythrocytes and higher RDW, and as a result, a rise in RDW could be a sign of aging erythrocytes, possibly as a result of a delay in erythrocyte clearance (25, 26). It has been demonstrated that oxidative stress can shorten erythrocyte survival time by damaging proteins, nucleic acids, and lipids, which in turn affects erythrocyte deformability and circulation half-life (27). According to Patel et al. proved that increased RDW and delayed erythrocyte clearance may be physiological reactions to stress and poor health (28). This could account for the link between RDW and mortality as well as its associated with poor prognosis across a wide range of patient populations. Additionally, oxidation increases nuclear factor kappa B (NF-B), which in turn promotes the release of cytokines like interleukin-6 (IL 6), in the inflammatory response (29). The inflammatory reaction can also hinder other biological processes. Both red blood cell maturation and the inflammatory response have the potential to be linked to increased RDW. Elevated RDW is linked to coronary artery disease, systemic atherosclerotic lesions, and even long-term prognosis in healthy people (7–11). RDW levels can be shown to be positively associated with bad disease outcomes and even illness outcomes in healthy populations, even if the exact mechanism by which elevated RDW is associated with mortality is still unclear, suggesting that RDW may be used as a predictor for screening for poor prognosis and, in combination with other risk predictors for disease, may be involved in the development of secondary prevention criteria.

Yazıcı et al. investigated the association between dynamic changes in red blood cell distribution width and 30-day mortality in 199 patients with acute pulmonary embolism (30). They discovered that increased levels of RDW were independently associated with mortality (HR: 4.9, (95% CI: 1.2–1.8, *p* = 0.02)) and were predictive of mortality in patients with acute pulmonary embolism. Ozsu et al. and Zhou XY et al. discovered comparable outcomes (31, 32). In 208 patients with pulmonary embolism who were evaluated for 100-day mortality, Sen et al. studied complete blood counts, markers of renal function, c-reactive protein, and the simplified pulmonary embolism severity index (sPESI) scoring system and discovered that RDW and sPESI may be reliable guidelines for predicting 100-day mortalit (33). A study by Savino Spadaro et al. found that red blood cell transfusion significantly increased RDW values and that blood transfusion may be an intervention to evaluate the prognostic role of RDW (34). However, Fogagnolo et al. demonstrated that higher RDW values at ICU admission were independently associated with 90-day mortality in critically ill patients, regardless of previous red blood cell transfusion (35). As our study used RDW values measured on the first day of admission, the effect of transfusion factors on the measurements was not significant. RDW was linked to long-term mortality in patients with pulmonary embolism in studies conducted by Kheirkham-Sabetghadam et al. and Zorlua et al. who prospectively evaluated a total of 136 consecutive patients with acute PE (36, 37). High RDW was linked to worse hemodynamic parameters and contributed to early risk stratification of patients with acute pulmonary embolism. There was a separate organization.

In a related investigation, Jingxue Pan et al. assessed RDW in 27,063 Cancer Cohort individuals between the ages of 45 and 73. Cox proportional risk regression analysis was performed to assess the relationship between RDW and all-cause and cause-specific mortality after 19.8 5.5 years of follow-up, while controlling for covariates. Nine thousand three hundred eighty-eight fatalities in all took place throughout the follow-up period (38). High RDW was substantially linked with cancer mortality, CVD mortality, respiratory disease mortality, and all-cause mortality. They propose conclude that RDW is associated with mortality and suggest that high RDW is an important but non-specific marker of mortality risk in the general population.

One of the frequent blood tests is the RDW value, which is frequently utilized in clinical practice. RDW values are frequently employed in the differential diagnosis of anemia, and in recent years, mounting evidence has shown that these values are linked to a variety of human diseases and their consequences, and more significantly, to overall mortality in the general population. Most researchers agree that expanding the clinical application of RDW to include erythrocyte fragmentation, poor nutritional status, hypertension, dyslipidemia, and erythropoietin abnormalities is more appropriate than restricting the traditional application of RDW to the early detection of anemia (39). In order to accurately forecast the prognosis of patients with a variety of common acute and chronic diseases, RDW can reflect general health condition as well as subclinical and clinical disease status (40, 41). The use of RDW as a stand-alone prognostic marker may be inadequate and that it may need to be combined with other clinical parameters to be truly useful in predicting the prognosis of critically ill patients (42). RDW is not currently included as an indicator in the SOFA score. Although RDW may act as a predictor in some cases, its predictive value in the SOFA score remains controversial and is not currently included in the SOFA score. Therefore, when assessing the clinical status of a critically ill patient, physicians may need to incorporate other clinical indicators and laboratory findings to fully assess the severity of the patient’s disease and prognosis. In future studies, there is continued interest in the potential benefits of adding the RDW to the SAPS or SOFA score as a prognostic tool for critically ill patients. This may help to inform treatment decisions and improve the prognosis of critically ill patients.

In conclusion, current research indicates that RDW is strongly correlated with a number of clinical diseases, however it is unknown how these changes are brought about. RDW would undoubtedly make it simpler to oversee clinical activity if it were employed as a prognostic monitor for common clinical disorders.

Weakness: (1) The majority of the adult patients in our unselected group were middle-aged or older, and variations in RDW levels may have been caused by the older population’s higher prevalence of medical problems and usage of various drugs. (2) Our research was cohort-based. There are other factors that could affect our findings on the association between RDW and mortality. (3) We are unable to link laboratory data to other potentially confounding variables, such as past history and treatment history. (4) Although we have adjusted for chronic conditions that may affect the results, we do not have information on cases of death due to complications from drug use in patients. (5) We do not know if there was active bleeding that might have influenced measurement results or if these unselected critically ill patients had blood transfusions before blood biochemistry testing.

Despite these constraints, the following features of our study are present: (1) Our cohort study’s sample size is substantial, and the sample is highly representative. (2) To gather all the data for analysis, our clinical laboratory follows a standard technique. (3) All significant confounders, including hemoglobin, the erythrocyte pressure product, red blood cells, white blood cells, etc., are adjusted for. (4) The MIMIC-III database has a sizeable and trustworthy sample. (5) The results of our investigation revealed a non-linear relationship between RDW and all-cause mortality in unselected critically ill patients, which has important ramifications for the use of illness markers in the future to help with mortality prediction. (6) One potential unique aspect of our manuscript is the longitudinal analysis, which refers to an investigation where participants and RDW are collected at multiple different follow-up timestamps (30-day, 90-day, 365-day, and 4-year). (7) Compared with previous studies, we not only studied the long-term mortality over 4 years but also used cubic splines to make the results more intuitive.

## 5. Conclusion

In unselected critically ill patients, RDW levels were positively associated with all-cause mortality, with elevated RDW levels increasing all-cause mortality. Our data show a non-linear relationship between RDW and all-cause mortality in unselected critically ill patients after adjusting for other confounders. All-cause mortality in critically ill patients is strongly correlated with RDW values, which may be a risk factor for patient death in the intensive care unit.

## Data availability statement

Publicly available datasets were analyzed in this study. This data can be found at: https://mimic.mit.edu/.

## Ethics statement

The Massachusetts Institute of Technology (Cambridge, MA) and Beth Israel Deaconess Medical Center (Boston, MA) approved the database’s establishment, and consent was acquired for the first data gathering. Hence, this research was exempt from the ethical approval statement and the necessity of informed consent.

## Author contributions

SP was in charge of the research’s overall execution and manuscript writing, while WK and WL were in charge of analyzing the data. All authors have accepted responsibility for the entire content of this manuscript and approved its submission.

## Funding

There was no particular funding for this research, but as part of the author’s work, the first author is a graduate student in the University of South China, Hengyang, Hunan Province, China.

## Conflict of interest

The authors declare that the research was conducted in the absence of any commercial or financial relationships that could be construed as a potential conflict of interest.

## Publisher’s note

All claims expressed in this article are solely those of the authors and do not necessarily represent those of their affiliated organizations, or those of the publisher, the editors and the reviewers. Any product that may be evaluated in this article, or claim that may be made by its manufacturer, is not guaranteed or endorsed by the publisher.

## Footnotes

^1^ http://www.r-project.org

^2^ http://www.empowerstats.com
